# Time heterogeneity of the Förster radius from dipole orientational dynamics impacts single-molecule Förster resonance energy transfer experiments

**DOI:** 10.1103/physrevresearch.7.023014

**Published:** 2025-04-04

**Authors:** David Frost, Keisha Cook, Hugo Sanabria

**Affiliations:** 1 School of Mathematical and Statistical Sciences, Clemson University, Clemson, South Carolina 29634, USA; 2 Department of Physics and Astronomy, Clemson University, Clemson, South Carolina 29634, USA

## Abstract

Förster resonance energy transfer (FRET) is a quantum mechanical process governing the nonradiative energy transfer between coupled electric dipoles. Its strong distance dependence makes it a widely used as a “molecular ruler” in biology, chemistry, and physics. In single-molecule FRET (smFRET) experiments employing time-resolved confocal microscopy, deviations from the theoretical Förster relationship between FRET efficiency and donor fluorescence lifetime—termed dynamic shifts—provide insight into underlying molecular conformational dynamics. A key challenge in interpreting these shifts is disentangling contributions from the intrinsic motion of the fluorescent dyes from those of the biomolecular system under study. We present a novel theoretical framework based on Langevin dynamics to model the stochastic translational and rotational motion of dye linkers, incorporating first-principles physics and chemical constraints consistent with molecular dynamics simulations. Our results demonstrate that the dominant factor influencing dynamic shifts in smFRET is the relative orientational fluctuations of the dyes’ electric dipole moments, rather than their accessible spatial volumes. These findings refine the theoretical foundations of FRET and provide the most precise estimates of FRET efficiency to date, enhancing its utility as a molecular-scale probe of dynamic processes.

## I. INTRODUCTION

Förster resonance energy transfer (FRET) is a widely used spectroscopic technique in biophysics and structural biology for probing molecular distances and conformational states at the nanometer scale [1–7]. FRET occurs through nonradiative energy transfer between a donor and an acceptor fluorophore, facilitated by electric dipole coupling. The efficiency of this transfer depends strongly on the donor-acceptor distance, scaling with the inverse sixth power of separation, as well as on dipole orientation and other time-independent factors [1,5,8–10]. This distance dependence enables FRET to function as a “molecular ruler” [3, 11]. However, direct interpretation of FRET-derived distances in terms of molecular structure is nontrivial due to various complicating factors, including fluorophore mobility, dynamic heterogeneity, and dye-specific effects [8,12–15]. While single-molecule FRET (smFRET) has become a key tool for resolving biomolecular conformational dynamics [11,16–34], precise understanding of FRET measurements is critical for applications such as biosensor development, signal transduction studies, and fluorescence-based drug discovery [35,36].

FRET can be analyzed via steady-state photon counting or time-resolved fluorescence measurements [37]. We and others have previously shown that the joint distribution of FRET efficiency and donor fluorescence lifetime provides deeper insight into molecular dynamics beyond what either quantity alone can reveal [31,38,39]. In particular, deviations from the ideal Förster relationship—termed “dynamic shifts” [38]—can encode structural fluctuations of biomolecules, with multiple studies illustrating how dynamic shifts refine our understanding of biomolecular conformational landscapes [21,30–32,40,41]. However, an inherent challenge in smFRET experiments is that the donor and acceptor fluorophores are not rigidly fixed; rather, they undergo thermally driven stochastic motion. These fluctuations modulate the joint FRET-lifetime distribution [39,42], making it essential to quantitatively account for their effects [23,43–46]. Without a comprehensive model for these fluctuations, FRET-derived molecular distances and conformational dynamics remain subject to uncertainty [47,48].

Existing theoretical models of dye motion introduce substantial uncertainties in FRET-based distance measurements [39,42]. Current approaches range from oversimplified isotropic models [49] to computationally expensive all-atom molecular dynamics (MD) simulations [20,48,50,51]. While MD simulations are often used to estimate the accessible volume of dyes and provide uncertainty quantification for FRET-lifetime distributions [32,48,52,53], they suffer from practical limitations: typical simulations do not capture the full temporal sampling relevant to smFRET experiments [48], and equilibrium-based approaches neglect time-dependent heterogeneity in dye motion. Consequently, the full range of dye dynamics remains unresolved.

In this work, we develop a semianalytical model of fluorescent dye motion to address three key questions. (1) Can an isotropic Gaussian process adequately describe dye motion? (2) How does linker length influence FRET measurements? (3) What is the role of dipole orientational dynamics in FRET efficiency fluctuations? Using simulated smFRET experiments, we show that the conventional assumption that dynamic shifts arise solely from dye translational motion [31,38] is incomplete. Instead, we demonstrate that dynamic shifts depend on the full-state dynamics of the dyes, encompassing both translational and rotational degrees of freedom. This finding has critical implications for reducing uncertainties in FRET-based molecular distance estimates and resolving biomolecular conformational dynamics. By leveraging dynamic shift signatures, our framework enables the decoupling of dye-specific motion from the intrinsic dynamics of the biomolecular system under study, advancing the precision and interpretability of smFRET experiments.

### A. Time-resolved confocal smFRET

Time-resolved confocal smFRET experiments provide a powerful tool for probing molecular structure and dynamics at the nanometer scale. By attaching fluorescent donor and acceptor dyes to a molecule of interest, exciting the donor, and measuring the resulting fluorescence emission, one can estimate the FRET efficiency and, in turn, infer molecular distances.

FRET efficiency estimation is typically performed using two complementary approaches: intensity-based FRET and lifetime-based FRET [5,54]. Both methods are applicable in time-resolved confocal smFRET experiments, and understanding their interrelationship is a central focus of this work.

In a typical experiment, the sample is diluted such that, on average, fewer than one molecule of interest resides within the confocal volume of the microscope at any given time [5,54]. As molecules freely diffuse through the confocal volume, donor fluorophores undergo repeated excitation cycles, and the emitted fluorescence is recorded. The transient residence of a molecule within the confocal volume results in a burst —a rapid sequence of detected photons, referred to as the burst time. By analyzing the histogram of the time delay between laser excitation and photon detection, one obtains a lifetime measurement, which reflects how long the donor remains in the excited state before returning to the ground state via fluorescence, FRET, or other relaxation pathways [54].

Each burst contributes a statistical sample of photon arrival times and fluorescence lifetimes, enabling the construction of the joint FRET-lifetime distribution. During a burst, the donor molecule undergoes repeated excitation cycles, where each cycle—termed an excitation event—results in either direct fluorescence emission, energy transfer via FRET, or nonradiative relaxation. Importantly, a single burst yields multiple photons, each contributing to the overall FRET-lifetime distribution, whereas an individual excitation event provides information about the spectral window of detection and decay time of the detected photon.

By systematically analyzing these bursts and their associated excitation events, one can disentangle molecular conformational dynamics from the stochastic fluctuations inherent in smFRET measurements. This study aims to elucidate the interplay between intensity-based and lifetime-based FRET, refining the theoretical framework for interpreting time-resolved smFRET experiments.

### B. FRET model

Consider two completely static dyes with normalized dipole moments ${\overset{ˆ}{\mu }}_{A}\in S^{2}$ and ${\overset{ˆ}{\mu }}_{D}\in S^{2}$ for the acceptor and donor, respectively. Further, let the interdye displacement vector be $r\in R^{3}$ and magnitude r=‖r‖. The energy transfer rate is defined in Eq. (1),

(1) $$k_{\text{ET}}(r)=k_{D}{\left(\frac{R_{0}}{r}\right)}^{6},$$

where $k_{D}$ is the radiative decay rate for the donor dye, and $R_{0}$ is the distance at which the energy transfer efficiency is 0.5 [2,5,17,46]. Note that the energy transfer rate increases steeply as the distance decreases and inversely as the distance increases. However, no matter the distance, no energy transfer can happen if ${\overset{ˆ}{\mu }}_{D},{\overset{ˆ}{\mu }}_{A}$, and $\overset{ˆ}{r}$ are mutually orthogonal [55,56]. The Förster radius can be written as $R_{0}^{6}(t)=C\kappa^{2}(t)$ where C is a constant depending on the environment surrounding the dye. The parameter $\kappa^{2}(t)$ is the dipole orientational factor

(2) $$\kappa^{2}(t)=\left[{\overset{ˆ}{\mu }}_{D}(t)\cdot {\overset{ˆ}{\mu }}_{A}(t)\right)-3\left(\overset{ˆ}{r}\cdot {\overset{ˆ}{\mu }}_{D}(t)\right){\left(\overset{ˆ}{r}\cdot {\overset{ˆ}{\mu }}_{A}(t)\right]}^{2}$$

with $\overset{ˆ}{r}=\frac{r}{\left\|r\right\|}$ [57]. Since the dipole moments are known to reorient on timescales faster than the energy exchange rate [56,58], kappa square ($\kappa^{2}(t)$) is treated as time-dependent. This is in contrast to previous models wherein the dipole moment is chosen from the equilibrium distribution of the rotational diffusion [46,59,60]. In this model, the initial distribution of the dipoles is chosen according to the equilibrium distribution, but rotational processes evolve during the energy transfer. This is vital because the FRET efficiency cannot be evaluated in terms of an evaluation of the energy transfer rate at a specific time but rather as dependent on the history of the $\kappa^{2}$ process using the fact that the transfer times at a time T>0 of a nonhomogeneous CTMC are exponential with rate $\int_{0}^{T}k(s)ds$ [61–64] therefore the FRET efficiency at time T is given by

(3) $$ℰ\left(T\right)=\frac{\int_{0}^{T}k_{\text{ET}}\left(s\right)ds}{\int_{0}^{T}k_{\text{ET}}\left(s\right)ds+k_{D}T}.$$

In this way, the FRET efficiency process, ℰ(t), is non-Markovian. It is important to note that each excitation event’s fluorescence process will still be Markovian. As noted in [59], the interarrival time for the photon count process need not be exponentially distributed [65]. Therefore the photon arrival process cannot be seen as a time-homogeneous Poisson process in contrast to previous common assumptions [4,23,66–68]. Depending on the rate at which the dyes reorient, each vector may be treated as uniformly distributed on the unit sphere or a cone [59]. In this case, the average value of $\kappa^{2}$ is given by $\frac{2}{3}$ [1]. This is referred to as the dynamic averaging regime [56,57,59].

**FIG. 1.**
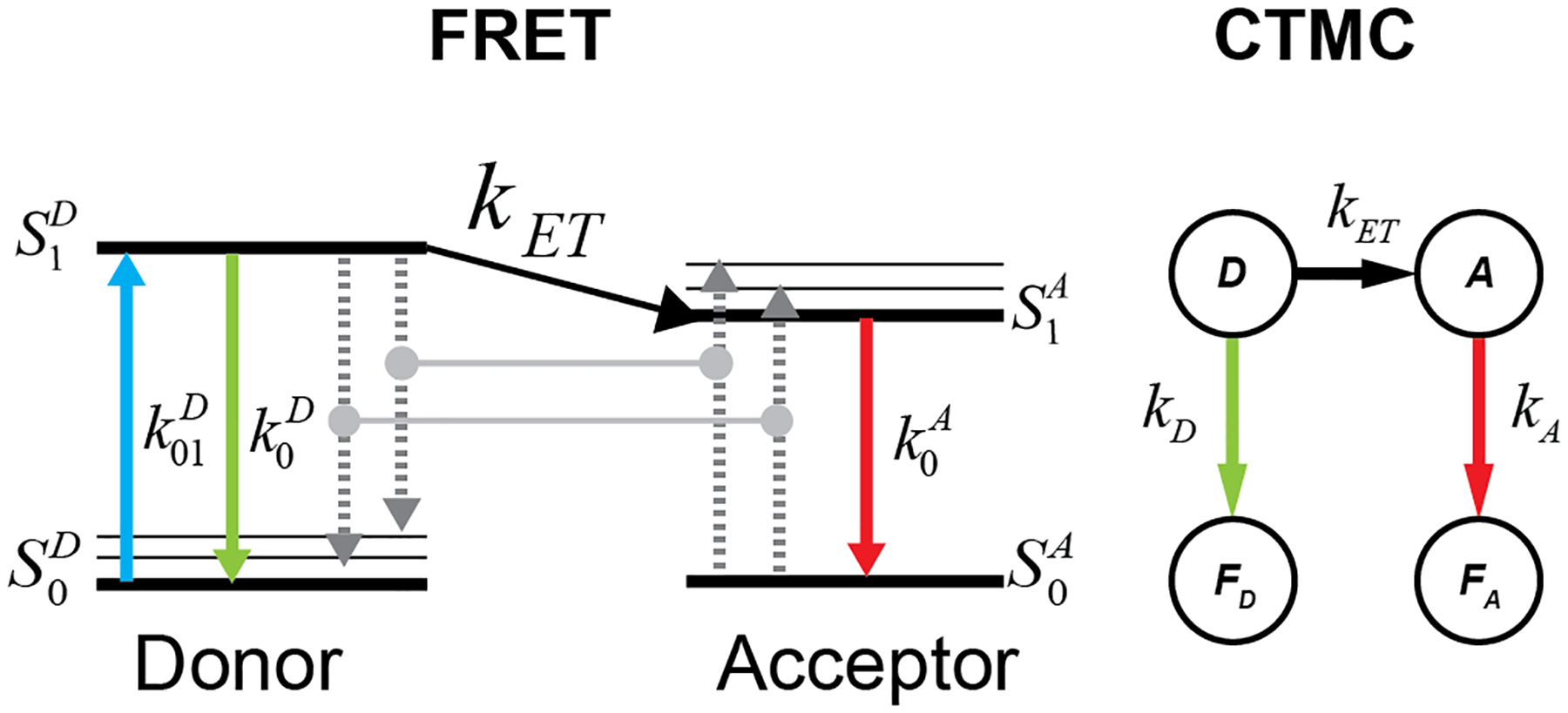
Representation of FRET by CTMC. Comparison of the CTMC states with the Jablonski diagram for the FRET process.

Using the fact that exponential random variables can well model fluorescence times [37] and accounting for the time dependence of the Förster radius on $\kappa^{2}$ the energy transfer process in FRET is modeled as a time-inhomogeneous continuous-time Markov chain (CTMC) [62,63], illustrated in Fig. 1, with the rate matrix defined in (4):

(4) $$Q(t)=\left(\begin{array}{cccc} -\left(k_{D}+k_{\text{ET}}(t)\right) & k_{\text{ET}}(t) & k_{D} & 0\\ 0 & -k_{A} & 0 & k_{A}\\ 0 & 0 & 0 & 0\\ 0 & 0 & 0 & 0 \end{array}\right),$$

where $k_{D}$ is the donor fluorescence rate, $k_{A}$ is the acceptor fluorescence rate, $k_{\text{ET}}$ is the FRET energy transfer rate. The state space is defined as $S=\left\{D,A,F_{D},F_{A}\right\}$, where D is the donor position, A is the acceptor position, $F_{D}$ is the donor fluorescence, and $F_{A}$ is the acceptor fluorescence.

Note that if r=0, the CTMC is reduced to a two-state system transitioning between states A and $F_{A}$ with rate $k_{A}$.

Assuming the dynamic averaging regime, one may easily derive the common time-homogeneous FRET efficiency. Observation of an acceptor photon only occurs when energy transfer occurs, i.e., if we have a transition from D→A. Let $\tau_{D}$ and $\tau_{\text{ET}}$ be the transfer times of $D\to F_{D}$ and D→A, respectively. Then using Eq. (3) one obtains

$$ℰ(t)=P\left(\text{min}\left(\tau_{D},\tau_{\text{ET}}\right)=\tau_{\text{ET}}\right)\;=\frac{\int_{0}^{t}k_{\text{ET}}(t)}{\int_{0}^{t}k_{\text{ET}}(t)+k_{D}t}\;=\frac{k_{D}\left(\frac{R_{0}}{r}\right)t}{k_{D}\left(\frac{R_{0}}{r}\right)t+k_{D}t}\;=\frac{1}{{\left(\frac{r}{R_{0}}\right)}^{6}+1}.$$

Therefore the theoretical time-homogeneous FRET efficiency is given by

(5) $$ℰ=\frac{1}{{\left(\frac{r}{R_{0}}\right)}^{6}+1}.$$

One may approximate this value using two methods: intensity-based FRET and lifetime-based FRET [46,54]. For *intensity-based* FRET, the measurements are counts of observed photons from each dye. Effectively, the experiment measures the probability of success of a binomial random variable with a probability of success p given by the FRET efficiency, ℰ. The best estimator in the absence of experimental corrections is given by the number of successes observed divided by the total number of trials, denoted in Eq. (6) [32,46,69–71];

(6) $${ℰ}_{I}=\frac{I_{A}}{I_{A}+I_{D}}.$$

For *lifetime-based* FRET, consider

$$ℰ+P\left(\text{min}\left(\tau_{D},\tau_{\text{ET}}\right)=\tau_{D}\right)=1.$$

Noting that since $P\left(\tau_{D}>t|\text{min}\left(\tau_{D},\tau_{\text{ET}}\right)=\tau_{D}\right)\sim \text{exp}\left(k_{D}+\right.k_{\text{ET}})$ [63], the FRET efficiency can be calculated in terms of the lifetimes,

(7) $$ℰ=1-\frac{\tau_{D}^{\prime }}{\tau_{D}},$$

where $\tau_{D}^{\prime }={\left(k_{D}+k_{\text{ET}}\right)}^{-1}$ is the lifetime of the donor in the presence of the acceptor, and $\tau_{D}=k_{D}^{-1}$ is the lifetime in the absence of the acceptor. Hence, the measurements are observed lifetimes and an estimate for the mean lifetime of the donor, $\tau_{D}$. The FRET efficiency is estimated by approximating the mean, and hence the rate, of this exponential random variable [54].

### C. Dynamic shift

Consider a sample drawn from a population with a distribution of fluorescence rates K(x) such that the probability of an individual having a specific rate is given by the distribution π(x). Then the average lifetime is

(8) $$\overset{‾}{\tau }=E\left[\tau \right]=\int_{R}E\left[\tau |K\left(x\right)\right]d\pi \left(x\right)=\int_{R}\frac{1}{K\left(x\right)}d\pi \left(x\right).$$

However, the lifetime resulting from the average rate is given by

(9) $$\underline{\tau }=\frac{1}{E[K(x)]}=\frac{1}{\int_{R}K(x)d\pi (x)}.$$

Therefore, by Jensen’s inequality [72], using the fact that $\phi (x)=\frac{1}{x}$ is convex for x∈[0,∞), it must be that

(10) $$\underline{\tau }=\frac{1}{E[K(x)]}⩽E\left[\frac{1}{K\left(x\right)}\right]=\overset{‾}{\tau }.$$

Consequently, the average lifetime for a mixture of states will be greater than that of the associated average state. This phenomenon is known as the dynamic shift.

**FIG. 2.**
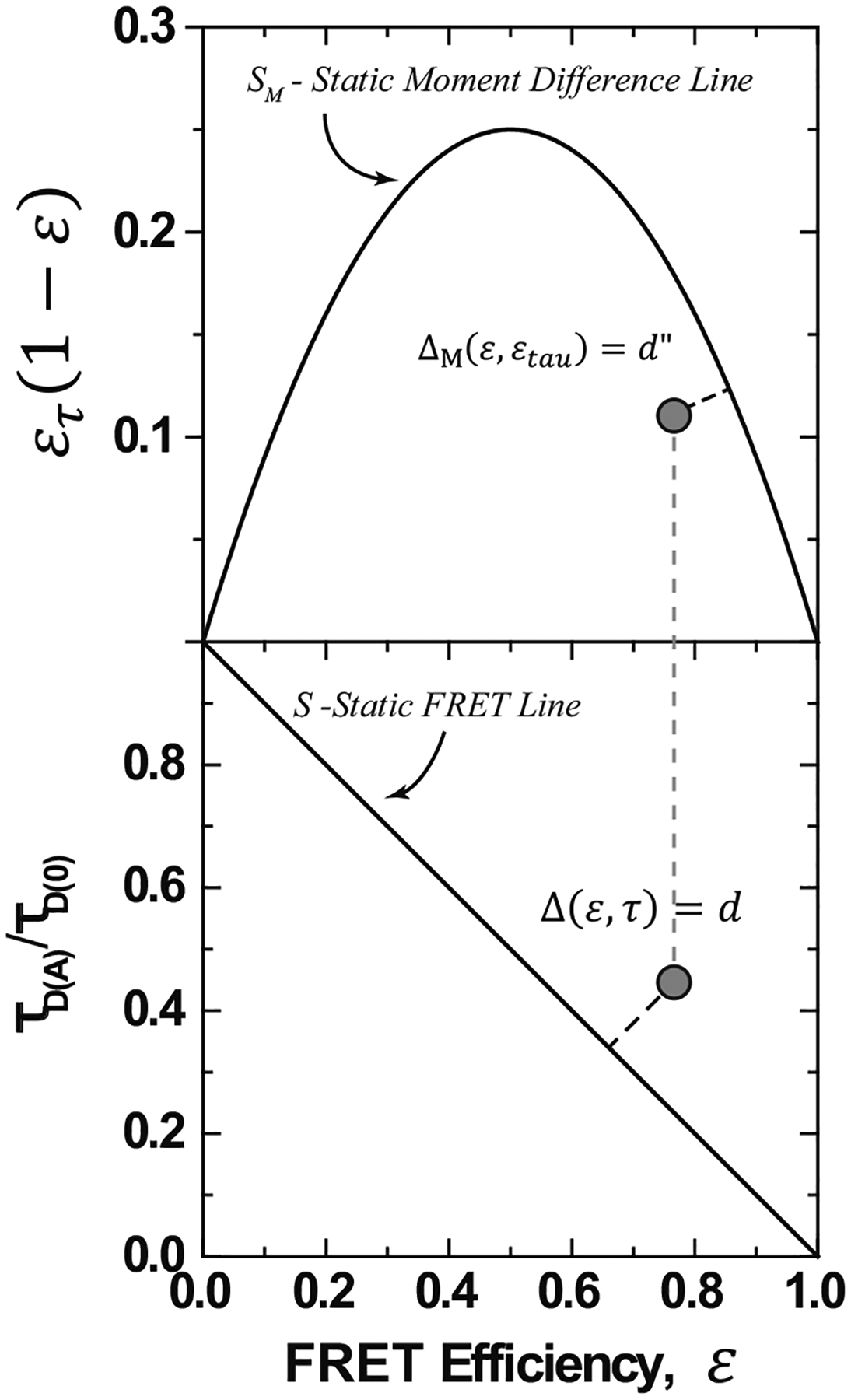
Visualization of the definition of the dynamic shift using normalized values: $S=\left\{(ℰ,\tau )\in (0,1{)}^{2}:ℰ+\tau -1=0\right\}$ in the bottom. The top figure is a visualization of the moment difference dynamic shift. For the moment difference we used ${ℰ}_{\tau }=1-\frac{\bar{\tau_{D(A)}}}{\tau_{D(0)}}$.

We introduce a new quantitative definition of the dynamic shift Δ for a point $\left({ℰ}^{\prime },\tau^{\prime }\right)$ in the plane, given by the signed distance from the point to the static line, S={(ℰ,τ):ℰ=1-τ} as shown in Fig. 2.

One can find an expression for the dynamic shift using standard analytic geometry. The formula in the normalized lifetime case, $\frac{\tau_{D(A)}}{\tau_{D(0)}}=\tau$, is given by

(11) $$\Delta \left(ℰ,\tau \right)=\frac{ℰ+\tau -1}{\sqrt{{ℰ}^{2}+\tau^{2}}}.$$

The definition of the dynamic shift becomes the signed length of the orthogonal projection of the point onto the static line—how much it deviates from the static line. Under the constraint that the FRET—lifetime pair resides within the unit square, this implies that the dynamic shift has extreme values at $\pm \frac{1}{\sqrt{2}}$ at (1,1) and (0,0). This definition provides a means by which each data point from a smFRET experiment may be assigned a dynamic shift value, and the resulting distribution may be examined. The average dynamic shift can be seen as an average deviation from the static line. With two state transitions, this definition agrees with the definition present in Ref. [38]. Furthermore, when the average dynamic shift is 0, one may use the dynamic shift distribution to quantify shot noise inherent in the measurements.

Another way to view the dynamic shift introduced in Ref. [38] is the moment difference approach. In this method, one investigates the behavior of the difference between the first and second moments of the FRET distribution, E[ℰ(1-ℰ)]=E[τ(1-τ)]. In this way, the effects of multiple states are linearized, while the static line is nonlinear. In this case, the dynamic shift can be seen as a consequence of Jensen’s inequality but for concave functions. When dynamic mixing is present in the sample, the moment difference should fall below the static line of ${ℰ}_{\tau }(1-ℰ)$. Note that when this difference is negative, it implies that the covariance between ${ℰ}_{\tau }$ and ℰ is larger than the average of ℰ. This can occur from shot noise or when the lifetime distribution has a large variance but maintains the same mean. Conditions for this to occur are discussed in Sec. III B. To define the dynamic shift from the static moment difference line, one again takes the distance from the point to the static line. The vector between the point and the static line with a length equal to the moment difference dynamic shift will be orthogonal to the tangent line of the static line at the point closest to the point.

The dynamic shift introduced in Ref. [38] considers an underlying distribution dependent on two separate states. Consider two FRET efficiency states denoted by ${ℰ}_{i},i=1,2$ with equal transition rate between the states λ for simplicity. Such a two-state system provides valuable insight into the nature of the dynamic shift. When two states are separated on long time scales, λ≪1, the dynamic shift is slight due to the small amount of mixing during a burst or sample. As the two states mix, corresponding to an increase in λ, an arc forms between the static FRET-lifetime coordinates, following $\left(1-{ℰ}_{1}-{ℰ}_{2}\right)ℰ-{ℰ}_{1}{ℰ}_{2}$. As λ→∞, this process culminates in a point mass FRET-lifetime distribution with a dynamic shift at the maximum of this arc. Therefore the dynamic shift can be seen as a metric of the amount of mixing between states. Two-state transition systems can be used to understand the transition rates between stable states in biomolecules conformational dynamics. For the current purpose, it provides a convenient method for interpreting the dynamic shift induced by the dyes. The dynamic shift will most readily be present when there exists mixing of states from a continuous state space. It will be shown in Sec. III that the dynamic shift induced by dye dynamics can be viewed as a consequence of the fluctuations in the energy transfer rate during the FRET process. Under common circumstances, the energy transfer rate can be approximated by a two-state system corresponding to the modes of the distribution, essentially leading to a quickly transitioning two-state system.

## II. STOCHASTIC MODELS OF FLUORESCENCE DYNAMICS

**FIG. 3.**
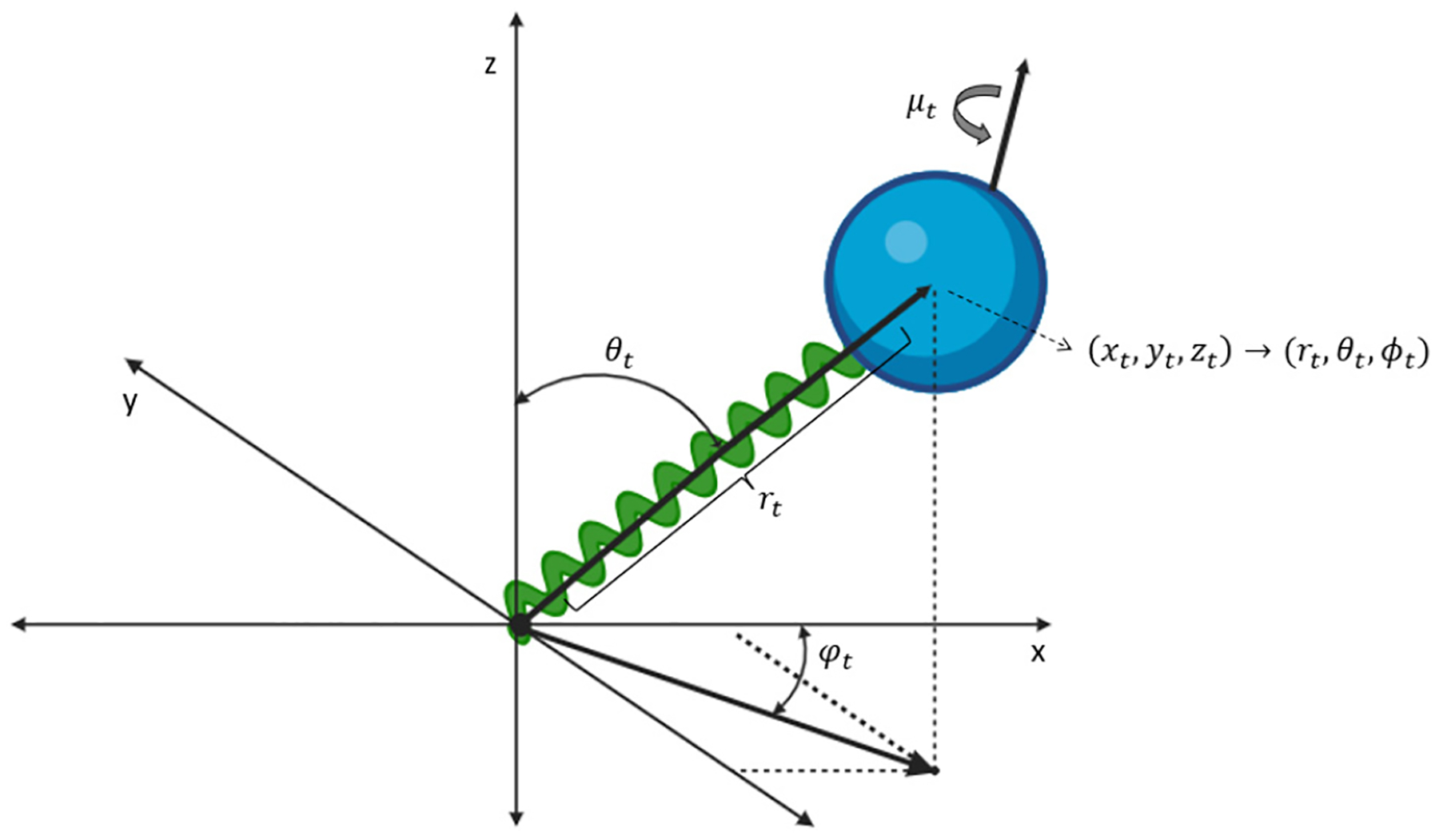
Cartoon showing the coordinate references for the processes. The translational process is expressed in both Cartesian and polar coordinates with $\phi_{t}$ the azimuthal coordinate, $\theta_{t}$ the polar coordinate, $r_{t}$ the radial coordinate, $\left(x_{t},y_{t},z_{t}\right)$ standard Cartesian coordinates, and $\mu_{t}$ the dipole orientational process.

This section presents several models of stochastic fluorescence dynamics related to the smFRET dynamic shift and associated molecular probes. In this way, estimation of the mixture of states, π(x) as seen in Sec. IC, is accomplished. Figure 3 shows the basic coordinate expression for the dye motion.

Throughout, the dynamics are assumed to evolve on different timescales. Letting $T_{P},T_{D},T_{O}$ represent the timescales of biomolecules dynamics, dye translational dynamics, and dipole orientational dynamics. The order of timescale separation assumed in this work is given by $T_{P}\gg T_{D}\gg T_{O}$. Further, as in Refs. [13,17,59,73,74], it is assumed that the orientation process and the translational process are independent processes. Note that this is an extremely common assumption since the independence of $\kappa^{2}$ and r dynamics is implicitly assumed whenever the average $\kappa^{2}$ value is used and whenever static $\kappa^{2}$ distributions are employed [75]. Moreover, to provide a clear and succinct picture of the influence of dye dynamics on FRET measurements, the timescale $T_{P}$ is not considered in the current discussion. However, an extension of this analysis to include this timescale is in development.

### A. Spring models

The simplest possible model to describe a stationary mean-reverting process is an Ornstein-Uhlenbeck (OU) process [76]. This physically represents an overdamped harmonic oscillator subject to noise [77]. The OU process is a Gauss-Markov process and, therefore, provides a simple model for thermal fluctuations of the fluorescent dyes. The equation of motion for the state vector $X_{t}\in R^{3}$ is given by the stochastic differential equation,

(12) $$dX_{t}=K\left(X_{t}-X_{\text{eq}}\right)dt+\sigma I\circ dB_{t},$$

where $K=k_{i,j}$ for i,j=1,2,3 is a matrix of spring constants and I is the identity matrix. The notation $\circ dB_{t}$ denotes the use of Stratonovich integration [78], where $B_{t}$ is Brownian motion. σ>0 is the volatility of the random fluctuations that are modeled as Brownian motions. We refer to systems such that the spring matrices can be written in the form $K=kI_{3\times 3}$, as isotropic springs. Otherwise, the system is called anisotropic.

Both isotropic and anisotropic spring systems with a diagonal spring matrix are considered. The spring coefficients are calculated using the linker chemistry. Utilizing the vibrational frequency of a C-C bond, we find that the spring constant for a single C-C bond is k=1010N/nm [79]. Therefore a system of N⩾1,C-C links is treated as a system of springs in series. Therefore

$$\frac{1}{k_{\text{eff}}}=\sum_{i=1}^{N}\frac{1}{k}\to k_{\text{eff}}=\frac{k}{N}.$$

Finally, to find the length of the linker, we investigate the equilibrium bond length, L, in a C-C-C link. Using the law of cosines, we find that $2L=\sqrt{2l^{2}-2l^{2}\;\text{cos}\;\theta }$ with l being the length of a C-C bond. Therefore the effective length in the linker for each link can be calculated using l=1.54Å and $\theta =109.5^{\circ }$.

**FIG. 4.**
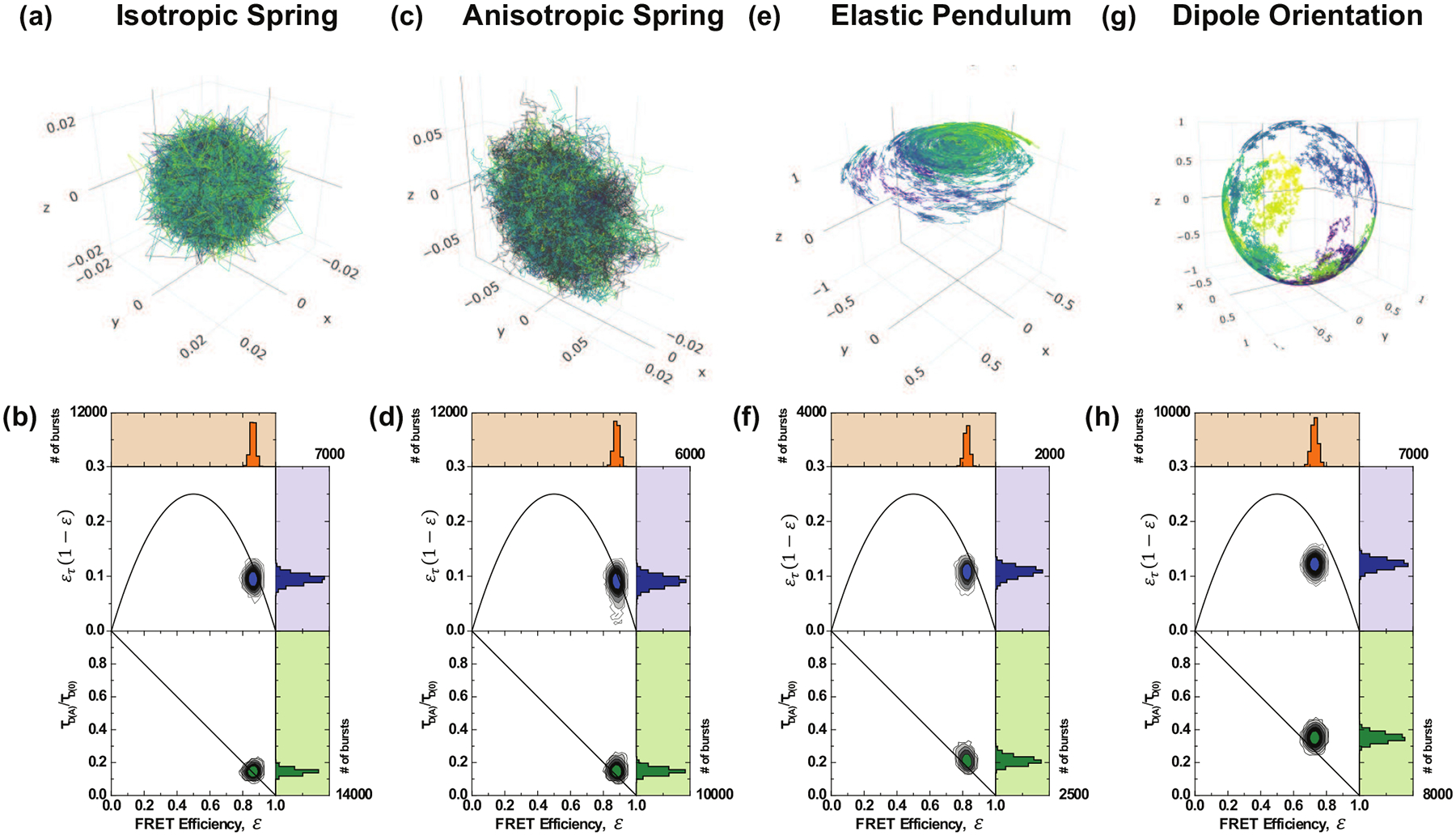
Dye model trajectories and resulting FRET efficiency vs normalized mean fluorescence lifetime and moments difference for [(a) and (b)] isotropic spring, [(c) and (d)] anisotropic spring, and [(e) and (f)] elastic pendulum, as described in Sec. II. [(g) and (h)] Sample trajectory of a spherical Brownian motion on the unit sphere $S^{2}$. Such processes are used to model the diffusion of the electric dipole moment. Each of (b), (d), (f), and (h) shows the FRET efficiency vs normalized mean fluorescence lifetime, and the moments difference when the dipole orientation for donor and acceptor is included in the FRET process. Each color in (a), (c), (e), and (g) is an individual burst trajectory. Each case simulates 25 000 trajectories over 7 hours. The values in (b), (d), (f), and (h) correspond to each simulated state.

In the isotropic case, illustrated in Fig. 4(a), the spring matrix is given by $k_{\text{eff}}I_{3\times 3}$. This provides a symmetric three-dimensional Gaussian as the stationary distribution for the isotropic spring [76]. It can be seen in Refs. [77,80] that the variance of this distribution will be given by $\Sigma =\frac{\sigma }{k_{\text{eff}}}I_{3\times 3}$.

In the anisotropic case, illustrated in Fig. 4(c), we use a diagonal spring matrix with two entries being $pk_{\text{eff}}$ and the third being $k_{\text{eff}}$ with p∈[0,1]. Therefore the stationary distribution is an ellipsoid with major axes determined by the entries of the spring matrix. In this section, rotational dynamics have not yet been considered; it will be covered in Sec. IIC.

The two-dimensional dynamics in the anisotropic case can be used to investigate the influence of the orientation of the stationary distribution on the resulting dynamic shift. Such a scenario is exemplified in the case when the planes formed by the major axes of each stationary ellipse are mutually orthogonal. Since the stationary distribution for the isotropic case is a sphere and is perfectly symmetric, this can only arise in the anisotropic case.

Furthermore, these models have the added benefit of having an analytical expression for the interdye displacement, especially in the isotropic case. Since the coordinates will be Gaussian distributed the distance between them is simply Rayleigh distributed [69]. This distribution is unimodal, and therefore the only mixing present is due to the variance of the stationary distributions. This mixing is therefore strongly dependent on the flexibility of the dyes.

### B. Elastic pendulum model

The next model for the dye linker dynamics takes a stochastic geometric mechanics approach. Consider the motion of a rigid body attached to a spring that is free to move in space. This system forms an elastic pendulum [81]. The following system of Langevin equations per component describes the motion of a point mass elastic pendulum system subject to white noise;

(13) $$dr_{t}=-k_{r}\left(r_{t}-r_{\text{eq}}\right)+\frac{1}{r_{t}}dt+\sigma_{r}\circ dB_{t}^{r}\;d\theta_{t}=-k_{\theta }\;\text{sin}\left(\theta_{t}\right)+\frac{\sigma_{\theta }^{2}}{r_{t}^{2}\;\text{tan}\;\theta_{t}}\circ dB_{t}^{\theta }\;d\phi_{t}=\frac{\sigma_{\phi }}{r_{t}\;\text{sin}\left(\theta_{t}\right)}\circ dB_{t}^{\phi }.$$

Similar to Eq. (12), $B_{t}$ is Brownian motion, σ>0 is the volatility of the random fluctuations that are modeled as Brownian motions, and $k_{r}$ and $k_{\theta }$ are the spring constant for the different components. Note that each superscript/subscript is indexed by each of the three components (r,θ,ϕ) explained in the next sentences. Importantly, the system is considered in spherical coordinates. The radial dynamics, $r_{t}$ evolve according to the spring dynamics explained in Sec. IIA, with slight alterations due to the change of coordinates. The angular parts of the motion are given by the standard nonlinear pendulum force in the polar direction $\theta_{t}$ and free diffusion in the azimuthal direction $\phi_{t}$. Figure 4(e) shows a sample dye trajectory.

The flexibility in the angular components is reminiscent of the wobble in a cone model used in previous investigations [49], and angular flexibility can be explained via the angular flexibility of C-C bonds themselves. However, unlike the classical wobble in a cone model, thermal noise and dye linker chemistry drive the dynamics and present a purely stochastic system. Moreover, by varying the parameters used, the system shows various behaviors.

**FIG. 5.**
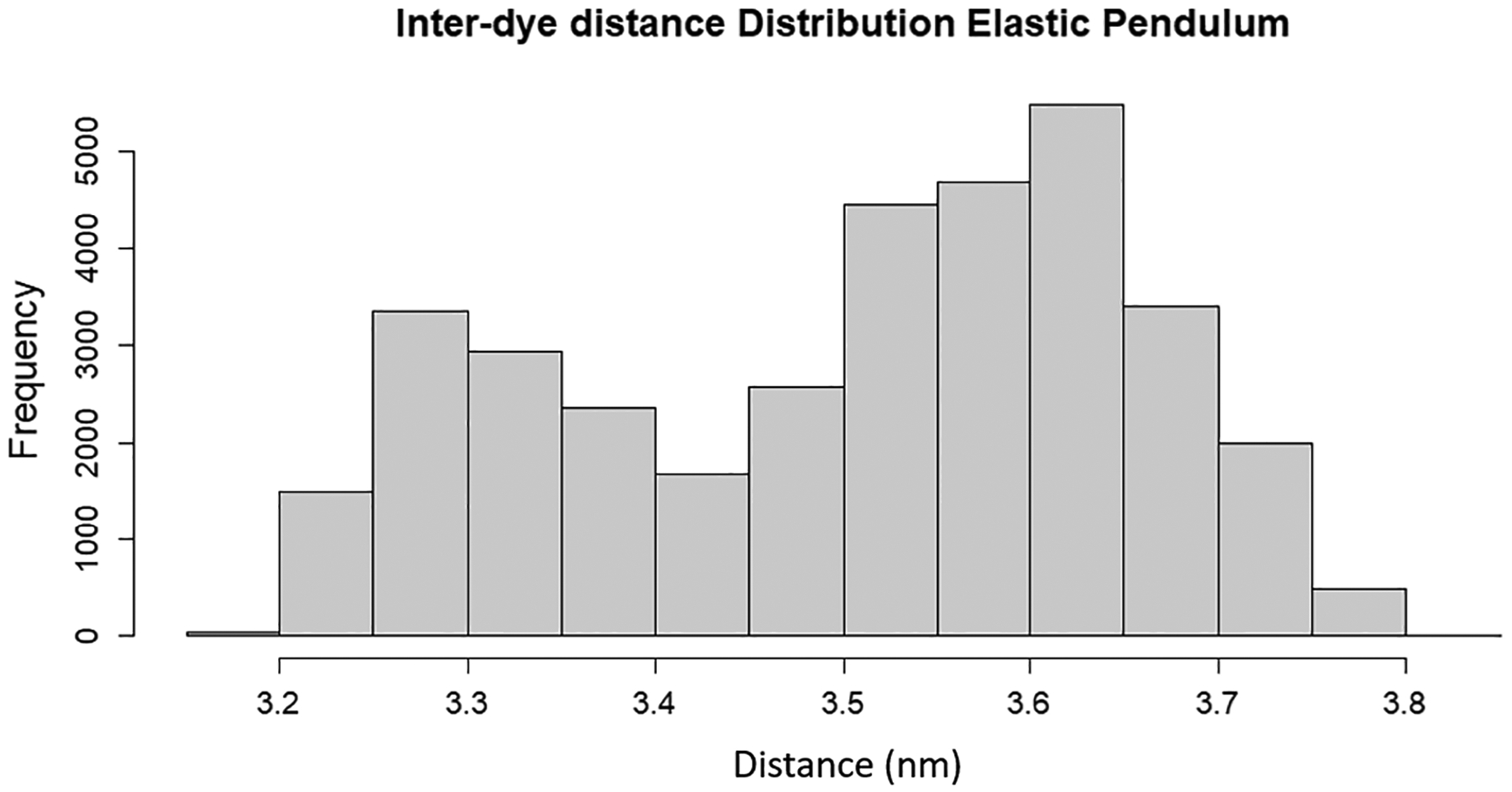
Histogram of interdye distances for the elastic pendulum model during an excitation event.

Moreover, this model presents a possible explanation for the dynamic shift induced by dye motion due to the non-Gaussian interdye distributions, as shown in Fig. 5.

This bimodality presents a mixing of two distinct states that are frequently needed to present a dynamic shift. Further, this provides a much larger change in interdye displacement than the spring models, which, as mentioned in Sec. IIA, do not possess any strong range of translational motion.

### C. Orientational dynamics

The final consideration involves the orientational dynamics of the electric dipole moments of the dyes. As discussed in Sec. I, the Förster radius is dependent on $\kappa^{2}$, which is dependent on the mutual orientations of the electric dipole moments ${\overset{ˆ}{\mu }}_{A}$ and ${\overset{ˆ}{\mu }}_{D}$ and the interdye displacement unit vector R. Typically, $\kappa^{2}$ is taken as the mean value of 2/3 when the unit vectors are considered uniformly distributed on the sphere $S^{2}$ [55,56,75]. This assumption ignores the temporal aspect of the fluorescent process. Since dyes reorient on timescales faster than fluorescent lifetimes, the energy exchange rate changes during the FRET process. This changes the original CTMC model to a time-inhomogeneous CTMC, and thus, the transfer rates are dependent on the time integral of the infinitesimal transfer rates [61–64].

To incorporate the influence of orientational dynamics on the lifetime distribution and FRET efficiency, consider the dipoles to be fixed to a reference frame of some rigid body with tensor of inertia I. The rigid body of the dye will be subjected to random torques and, therefore will reorient according to the Euler equations [81,82]

(14) $$Id\omega_{t}+\omega_{t}\times I\omega_{t}=-v\omega_{t}+dW_{t}\;\omega_{t}=d{𝚽}_{t},$$

where $\omega_{t}$ is angular velocity, ν is the dynamic viscosity of the surrounding fluid, $dW_{t}$ is a spherical Brownian motion and ${𝚽}_{t}$ is the angular position vector. Assuming the dye is overdamped and hence $d\omega_{t}=0$, one obtains the simplified equations

(15) $$\omega_{\text{t}}\times I\omega_{\text{t}}=-v\omega_{\text{t}}+dW_{\text{t}}\;\omega_{\text{t}}=d{𝚽}_{\text{t}}.$$

Making the assumption that the dye is spherical and therefore the inertia tensor may be replaced with a scalar value [81] and using the fact that v×v=0 for any vector v we obtain the simple formula

(16) $$vd{𝚽}_{t}=dW_{t}$$

and hence, the dipole diffuses according to a spherical Brownian motion. Spherical Brownian motion components can be expressed in terms of the Langevin equations below [83,84]

(17) $$d\theta_{t}=\frac{\sigma_{\theta }^{2}}{\text{tan}\left(\theta_{t}\right)}dt+\sigma_{\theta }\circ dB_{t}\;d\Phi_{t}=\frac{\sigma_{\phi }}{\text{sin}\;\theta_{t}}\circ dB_{t}.$$

The rotational diffusion coefficients depend on the hydrodynamic radius of the dye $R_{h}$ by the classical relation $D=kT/\left(8\pi \nu R_{h}^{3}\right)$, where kT denotes the product of the Boltzmann constant and the temperature. A sample trajectory is shown in Fig. 4(g).

Note that the stationary distribution for such a system is the uniform distribution, providing an ideal starting stochastic process to test the time-dependent behavior of orientational dynamics [78,82]. The key idea is that the excitation of the fluorophores provides a single initial $\kappa^{2}$ value. The relaxation effects are the object of interest, especially with regard to lifetime duration. The notion that $\kappa^{2}$ may be close to 0 during the entire FRET process for one excitation but higher for another in the same sampling time provides an additional source of variance in the lifetime distribution. Every FRET process can lead to a different equilibrium, which should depend on the rotational diffusion of the dipole moment, with faster reorientation causing an averaging out effect as mentioned in Ref. [56].

## III. SOURCES OF OBSERVED DYNAMIC SHIFT

### A. Dye configuration

This section compares the dye models described in Sec. II. By examining the joint FRET-lifetime distributions through the contour plots and marginal histograms in Fig. 4, the influence of the different models becomes evident. These FRET-lifetime distributions were generated by simulating the aforementioned models within a time-resolved confocal smFRET environment. As shown, the spring models exhibit characteristics similar to the anisotropic model, although the latter displays slightly more dynamic shifting. Additionally, the elastic pendulum model produces a noticeable dynamic shift, with the bulk of the distribution deviating from the static line. Despite the dynamic mixing inherent in the purely translational elastic pendulum model, the resulting dynamic shift does not fully capture the behavior previously observed in experimental data [7]. It is only with the incorporation of orientational motion and time-inhomogeneous energy transfer rates that the distribution exhibits the hallmark dynamic shift, both in terms of moment differences and within the direct FRET-lifetime distribution.

**FIG. 6.**
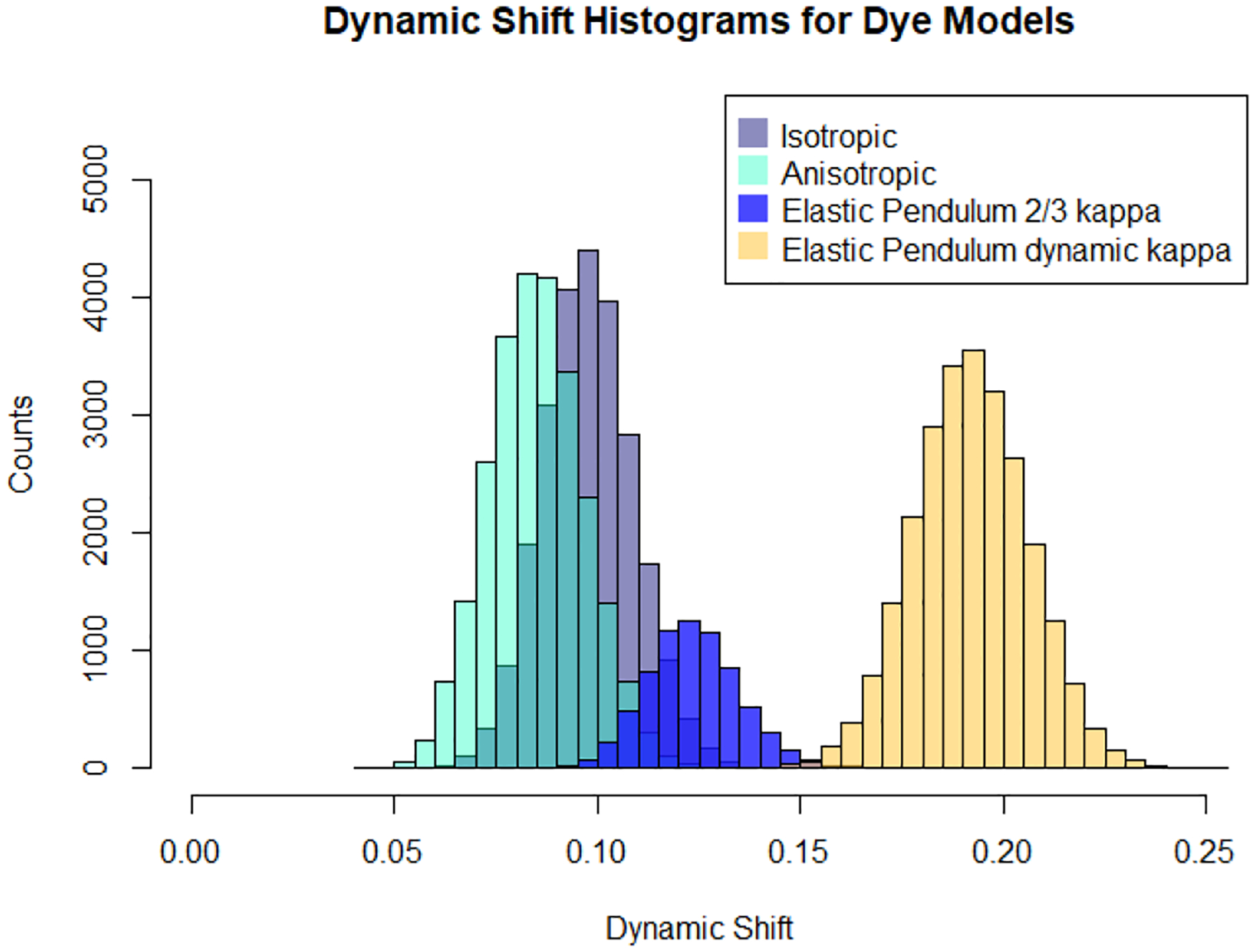
Linear dynamic shift, d, histogram comparison of dye models.

The better visualize these observations, we calculated the dynamic shift distributions of each dye configuration using the definition of the dynamic shift shown in Eq. (11). It has been known from experimental data that the average dynamic shift of dye motion is μ(Δ)≈0.2 [38]. Using this quantity, the average dynamic shift for the associated models is examined to determine the model that captures the appropriate mean dynamic shift. The comparison of the dynamic shift distributions is shown in Fig. 6.

In addition, these simulations have no burst noise from background radiation, as this could potentially cloud the impact of the dye motion [68]. The noise is solely from the experimental photon loss considerations and the dye motion as dictated by the models and simulation methods. Therefore the only source of dynamic shifting must be from the dye models.

Curiously the dynamic shift densities shown in Fig. 6 exhibit similar variances but differing mean dynamic shift values, as seen in Table I.

While the elastic pendulum model does not fully capture the mean dynamic shift, the incorporation of a dynamic $\kappa^{2}$ parameter results in the emergence of the expected dynamic shift. This finding contrasts sharply with previous hypotheses attributing the dynamic shift solely to the accessible volume of the dye. Despite the elastic pendulum model exhibiting an accessible volume comparable to that observed in all-atom molecular dynamics (MD) simulations and demonstrating dynamic mixing between conformational states, the resulting dynamic shift remains smaller than anticipated. This discrepancy suggests that time inhomogeneities in the Förster radius, driven by orientational fluctuations, are critical for accurately describing the dynamic shift.

It is noteworthy that spring models can, in principle, be modified to include a dynamic $\kappa^{2}$ parameter. However, when model parameters are constrained by the physical properties of linker compositions, these models become impractical. Specifically, the resulting accessible volumes are significantly smaller than what is physically reasonable. This limitation is evident when analyzing the variance of the stationary distributions associated with these models. As described in Sec. IIA, the variance along each axis in the isotropic case is given by $\sigma /k_{\text{eff}}$ or equivalently $k_{B}T/\gamma k_{\text{eff}}$, where γ represents the local friction coefficient. Under these conditions, the stationary distribution yields a 3σ radius of less than one angstrom, which is inconsistent with previously observed dye dynamics.

### B. $\kappa^{2}$ dynamics

An important consideration highlighted in Sec. IIIA is the role of $\kappa^{2}$ dynamics in shaping FRET-lifetime correlations. A central challenge in incorporating $\kappa^{2}$ dynamics into FRET uncertainty quantification lies in the common assumption that $\kappa^{2}$ remains stationary during the energy transfer process. This assumption is often addressed by sampling $\kappa^{2}$ from its equilibrium distribution, modeling it as a discrete-state Markov chain, or employing the conventional $\left\langle \kappa^{2}\right\rangle =2/3$ approximation [59,85–87]. Such approaches facilitate the use of mean and standard deviation estimates in uncertainty analysis [55,56,75]. However, these approximations neglect the time-inhomogeneous nature of the FRET process, as discussed in Sec. IB. Notably, the probability of donor fluorescence emission depends on the integrated history of energy transfer rates, inherently linked to the temporal evolution of $\kappa^{2}$ [61,62,64,88].

Although ergodic assumptions may partially address these concerns, they rely on the equivalence of long-term time averages and spatial averages, an approximation that breaks down over the short timescales characteristic of FRET, particularly under conditions of slow rotational diffusion. Moreover, such assumptions overlook the path-dependent nature of energy transfer, where the FRET rate constant is influenced by the specific temporal trajectory of $\kappa^{2}$. In experimental settings, FRET bursts typically span milliseconds, corresponding to single-molecule events, whereas simulations often generate bursts at one-second intervals, yielding approximately 25 000 bursts for statistical analysis.

**FIG. 7.**
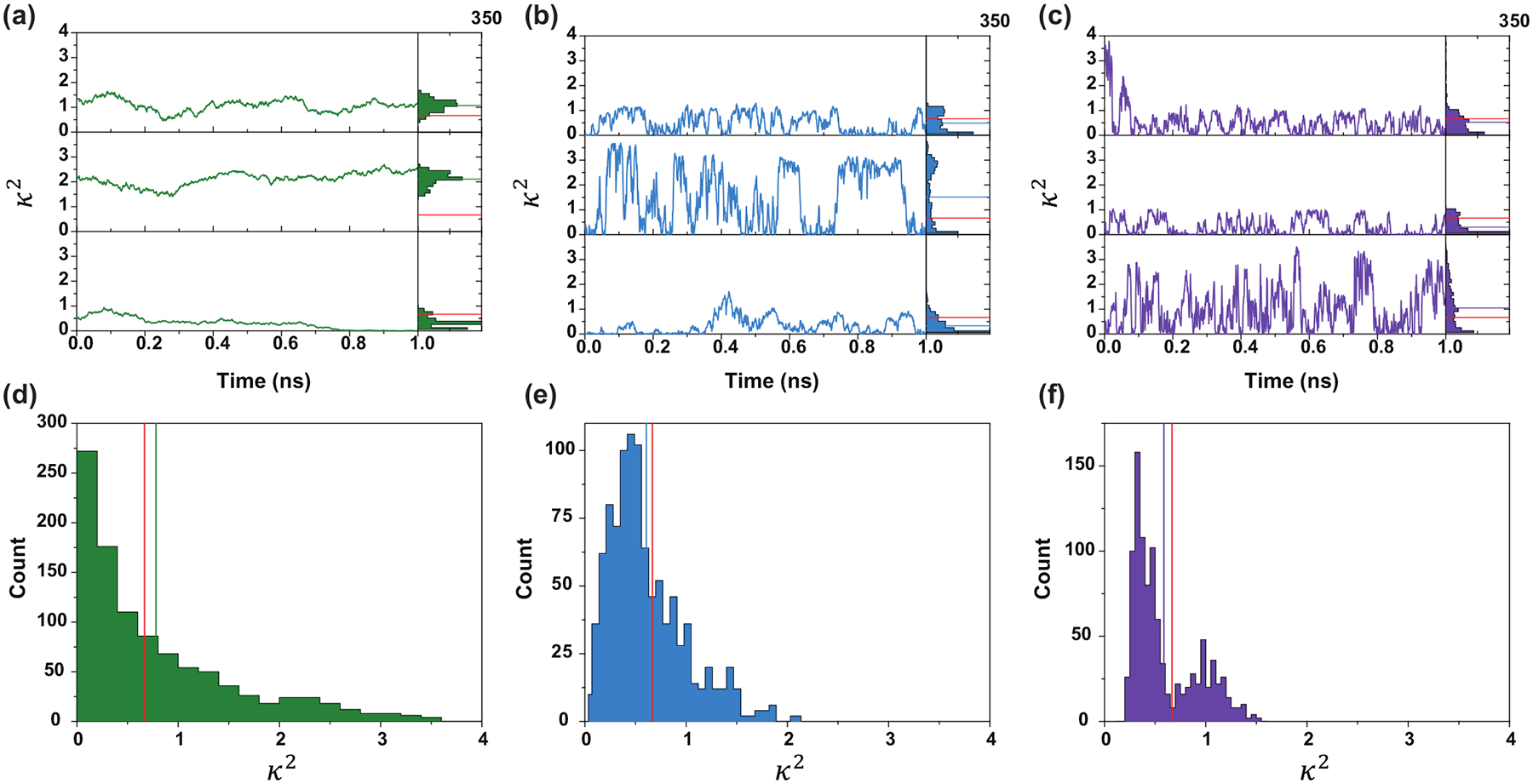
Comparison of $\kappa^{2}$ trajectories for three common cases. (a)–(c) shows the sample paths of $\kappa^{2}$ during an excitation event, these can be seen as four realizations of $\kappa^{2}$ during the same burst. Each path is representative of a single energy transfer event. (d)–(f) show the associated distribution of average $\kappa^{2}$, the red vertical lines in (d)–(f) are the isotropic 2/3 average, where the colored lines are the mean of the path average. (a) and (d) show a pair of dyes for which the rotational diffusion is only an order of magnitude greater than the translational diffusion of the dye. (b) and (e) show the sample behavior when one dye has a rotational diffusion three orders of magnitude greater than translational and one dye one order of magnitude greater. Finally, (c) and (f) show the case in which both dyes have rotational diffusions three orders of magnitude greater than translational. Parameters used for the simulation are shown in Table II.

As illustrated in Fig. 7, the stochastic nature of $\kappa^{2}$ manifests prominently in its temporal trajectories. For instance, Figs. 7(a) and 7(b) display trajectories with similar mean values over a 1 ns timescale. However, at intermediate times (e.g., 0.5 ns), significant differences in the probability of energy transfer emerge, highlighting the sensitivity of FRET to transient fluctuations in $\kappa^{2}$. Rapid rotational diffusion, as shown in Fig. 7(c), induces frequent oscillations in $\kappa^{2}$, leading to tightly clustered trajectories. Interestingly, the corresponding average $\kappa^{2}$ distribution [Fig. 7(f)] exhibits bimodality, reflecting populations of both low and high $\kappa^{2}$ values. This bimodality arises from variations in the radial-dipole dot product, a key term in the definition of $\kappa^{2}$. Parameters used for these simulations are shown in Table II.

Conversely, reduced rotational diffusion slows $\kappa^{2}$ fluctuations, resulting in prolonged intervals of high or low $\kappa^{2}$ values. These extended periods influence donor lifetimes asymmetrically: high $\kappa^{2}$ stretches accelerate energy transfer, shortening lifetimes, while low $\kappa^{2}$ periods prolong them. Consequently, slower rotational dynamics introduce stronger temporal correlations, amplifying the influence of the stationary $\kappa^{2}$ distribution on FRET behavior.

The skew observed in the mean $\kappa^{2}$ distribution underscores the limitations of simple averaging assumptions. Although the ensemble-averaged $\left\langle \kappa^{2}\right\rangle$ equals 2/3, the most probable values often fall below this benchmark. This deviation is evident in different simulation conditions [Figs. 7(a)–7(f)], with only Fig. 7(e) showing a mode near 2/3, likely due to enhanced temporal fluctuations in $\kappa^{2}$.

The dynamic shift observed in FRET experiments thus emerges from the inherent variability of $\kappa^{2}$ trajectories. Temporal heterogeneity during energy transfer introduces critical mixing effects that substantially modulate the average donor lifetime. Although interdye distance fluctuations contribute modestly to this dynamic shift (Fig. 4), rotational dynamics exert a more pronounced influence by directly altering the FRET rate constant. Figures 7(d)–(e) represent average $\kappa^{2}$ values over individual bursts, revealing how bimodal distributions in fast-rotating dye systems create distinct energy transfer populations. This behavior parallels two-state models explored in previous studies [31,38].

However, it is essential to emphasize that pathwise heterogeneity is central to smFRET analysis. Although average $\kappa^{2}$ values provide useful summaries, they do not capture the full complexity of the time-dependent FRET efficiency ℰ(t), which varies along individual stochastic trajectories. The data presented in Fig. 7 spans only 1ns and thus does not encompass the complete temporal dynamics of fluorescence decay or energy transfer. Ultimately, it is the path-dependent fluctuations in $\kappa^{2}$ that shape the observed lifetime distributions.

## IV. DISCUSSION

In this work, we present a physics-based model for fluorescence dynamics that explicitly incorporates dye linker chemistry and fluorescent dye composition, revealing the critical role of time-inhomogeneous Förster radius fluctuations in driving the dynamic shift observed in single-molecule FRET (smFRET) experiments. Our findings demonstrate that traditional models employing static accessible volumes fail to reproduce experimentally observed dynamic shifts. Instead, these shifts arise from the time-dependent nature of the FRET process, governed by the path-dependent dynamics of $\kappa^{2}$ trajectories. Importantly, we show that the characteristics of $\kappa^{2}$ fluctuations are sensitive to the specific fluorescent dyes used. For example, pairs involving an organic dye and a fluorescent biomolecule exhibit markedly different dynamics compared to pairs of organic dyes. While the mean $\kappa^{2}$ remains consistent with the isotropic approximation of $\left\langle \kappa^{2}\right\rangle =2/3$, fluctuations in $\kappa^{2}$ during energy transfer events introduce temporal heterogeneity in FRET-lifetime distributions within individual bursts. These fluctuations act as a source of dynamic mixing, manifesting as the dynamic shift. Crucially, while FRET efficiency remains unchanged, the donor lifetime distribution is significantly affected.

Although our current results focus on spherical dyes, the rotational Langevin dynamics outlined in Sec. IIC can be generalized to dyes with arbitrary inertia tensors. While straightforward in principle, the resulting equations become increasingly complex, both analytically and computationally, as the symmetry of the system decreases. Nonspherical dyes introduce anisotropic rotational behavior, leading to distinct $\kappa^{2}$ trajectories that require advanced numerical approaches for accurate simulation.

An important direction for future research involves coupling translational and rotational dynamics. While the decoupling of these motions is a common simplification—frequently invoked in dynamic averaging of $\kappa^{2}$ and in theoretical models such as [59]—evidence suggests that this assumption may overlook significant correlations. As highlighted in Ref. [75], translational motion can strongly influence $\kappa^{2}$ dynamics. This coupling becomes even more evident when considering the combined effects of orientational rigid-body dynamics and elastic linker fluctuations, as discussed in Secs. IIB and IIC. Such systems are known to exhibit nontrivial coupling even in classical mechanics, and similar effects are expected in the overdamped regime relevant to smFRET. However, accounting for these interactions necessitates modeling the system’s full state space on the noncompact, non-Abelian Lie group SE(3), as described in [82], significantly increasing both analytical and computational complexity compared to models with independent translational and rotational processes.

Moreover, the framework developed here provides a foundation for exploring additional sources of dynamic shift. The algorithms employed for simulating FRET dynamics are computationally efficient and readily adaptable to systems with multiple interacting timescales. This flexibility enables investigations into the influence of biomolecular conformational dynamics on FRET signals. By incorporating reaction-coordinate Langevin models, future studies can examine how complex energy landscapes and dynamic interdye distance fluctuations modulate the dynamic shift. The stochastic nature of our simulations facilitates rapid exploration of such scenarios, offering a practical tool for probing both dye and biomolecular dynamics. Notably, while our simulations are based on confocal microscopy conditions, the results are equally applicable to total internal reflection fluorescence (TIRF) measurements, broadening the scope of potential experimental validations.

In conclusion, this work highlights the significance of time inhomogeneities in the FRET process and their measurable impact on fluorescence lifetime distributions. We demonstrate that the path dependence of FRET efficiency, coupled with a time-varying Förster radius, is non-negligible and must be accounted for in accurate smFRET data interpretation. Optimal uncertainty quantification for smFRET requires a detailed understanding of $\kappa^{2}$ path dynamics, beyond simple ensemble-averaged values. Consequently, the anisotropic rotational behavior of fluorescent dyes plays a pivotal role in FRET measurements. By advancing our understanding of dye orientational dynamics, we pave the way for more precise uncertainty quantification in FRET studies. Furthermore, this work highlights the need for stochastic geometric mechanics approaches in the analysis of fluorescence phenomena, with broader implications for biophysical measurements and molecular biology.

## Figures and Tables

**TABLE I.** Dynamic shift. Mean and standard deviation of the dynamic shift for each model in Fig. 6.

Model	μ(𝚫)	σ(𝚫)
Isotropic spring	0.10	0.01
Anisotropic spring	0.01	0.01
Elastic pendulum $\kappa^{2}\approx 2/3$	0.12	0.01
Elastic pendulum dynamic $\kappa^{2}$	0.20	0.10

**TABLE II.** Simulation parameters for Fig. 7.

Parameter	(a) and (d)	(b) and (e)	(c) and (f)
Donor rotational diffusion	15 nm^2^/s	15 nm^2^/s	200 nm^2^/s
Acceptor rotational diffusion	15 nm^2^/s	150 nm^2^/s	200 nm^2^/s
Translational rotation	0 nm^2^/s	0 nm^2^/s	0 nm^2^/s

## Data Availability

Code is available at GitHub [89]. No data were created or analyzed in this study.
